# Haemorrhagic pneumonia in sled dogs caused by *Streptococcus equi* subsp. *zooepidemicus* - one fatality and two full recoveries: a case report

**DOI:** 10.1186/1751-0147-55-67

**Published:** 2013-09-11

**Authors:** Gry Jaeger, Hege Kippenes Skogmo, Øyvor Kolbjørnsen, Hans Jørgen Søiland Larsen, Bjarne Bergsjø, Henning Sørum

**Affiliations:** 1 Norwegian School of Veterinary Science, Department of Companion Animal Clinical Sciences, PO Box 8146 Dep, N-0033 Oslo, Norway; 2 Norwegian Veterinary Institute, Department of pathology, Sentrum, PO Box 750, N-0106, Oslo, Norway; 3 Norwegian Veterinary Institute, Department of Bacteriology – Animals and Fish, Sentrum, PO Box 750, N-0106, Oslo, Norway; 4 Norwegian School of Veterinary Science, Department of food safety and infection biology, Section for microbiology, immunology and parasitology, PO Box 8146 Dep, N-0033 Oslo, Norway

**Keywords:** Respiratory infection, Canine, Rehabilitation, Treatment, Immunology, Histology

## Abstract

In spite of yearly vaccination, outbreaks of canine infectious respiratory disease are periodically seen amongst domestic dogs. These infections compromise host defense mechanisms, and, when combined with other stressful events, allow opportunistic pathogens like *Streptococcus equi* subsp. *zooepidemicus* to create serious disease. Early recognition and treatment are tremendously important for a successful outcome in these cases. A polyvalent vaccine was given to 22 racing dogs three days after a competition, followed by two days of rest, and then the dogs were returned to regular training. Coughing was noticed among the dogs four days after immunisation. Three days after this outbreak one of the dogs was unusually silent and was found dead the next morning. Simultaneously two other dogs developed haemorrhagic expectorate, depression and dyspnea and were brought in to the veterinary hospital. *Streptococcus equi* subsp. *zooepidemicus* was isolated in pure culture from all three cases. They were treated and rehabilitated successfully, and won a sledge race three months later. This paper discusses the necropsy results, treatment regime, rehabilitation and the chronology of vaccination, stressful events and disease.

## Background

Contagious upper airway infections in dogs occur regularly and are most commonly caused by canine parainfluenza virus (CPIV) or *Bordetella bronchiseptica*, amongst other agents [1]. This clinical syndrome has also been named infectious tracheobronchitis (ITB), canine infectious respiratory disease (CIRD), “kennel cough” or “kennel croup”, so named due to its occurrence in environments where many dogs live or stay close together for shorter periods of time. Characteristic clinical signs include a self-limiting paroxysmal cough lasting for up to two weeks, which usually resolves without treatment. In Norway, immunisation against CIRD is performed using live attenuated viruses, annually with CPIV and every third year against canine adenovirus type 2 (CAV-2) [1]. However, in spite of vaccination, outbreaks of CIRD remain common. Some dogs with CIRD will develop serious pneumonia due to an immature immune system or other causes of immunodeficiency. Occasionally, bacteria such as *Streptococcus equi* subsp. *zooepidemicus* can cause fatal pneumonia [2-8].

This case-report describes the first canine outbreak of haemorrhagic pneumonia in the Nordic countries caused by *S. equi* subsp. *zooepidemicus*. Most of the animals in the pack of athletic sled dogs showed symptoms of CIRD with three dogs demonstrating symptoms of severe peracute infection. One sled dog died while two were successfully treated, rehabilitated and returned to competition. To the author’s knowledge, this is the first report documenting the chronology from onset of clinical signs, through convalescence to complete recovery for peracute haemorrhagic pneumonia in dogs. The vaccination regimen related to season and extreme training will also be discussed.

## Case presentation

The affected animals belonged to a pack of 26 racing sled dogs with one owner. Twenty-two dogs where in active competition/training at an international level, competing annually in the Norwegian, European and World championships. The dogs were a mix of pure bred German shorthaired pointer (GSP), mixed-bred Alaskan husky/GSP or mixed-bred GSP/greyhounds. The dogs were mainly kept as outdoor dogs in standard heated kennels, but at times allowed indoor as family pets.

In October 2008 they participated in the Norwegian National Championship in 4-dogs and 8-dogs classes. Prior to the race all dogs were healthy with no signs of infection or disease.

Three days after the race the 22 competing dogs were vaccinated with Duramune DAPPi + LC vet. Fort Dodge containing attenuated distemper virus (CDV), strain Onderstepoort, attenuated adenovirus type 2 (CAV-2), attenuated parvovirus (CPV), attenuated parainfluenza virus (CPIV), inactivated enteric coronavirus (CCoV) as well as inactivated *Leptospira interrogans* serovar Canicola and serovar Icterohaemorrhagiae. After vaccination the dogs rested for two days with no exercise or training. On the third day the dogs continued regular training. Following training on the 4th day post-vaccine, several dogs in the kennel started to cough, and 24 hours later there were more animals in the pack with an intensive cough. All animals had normal body temperature and showed no other clinical signs. Six days after the vaccine two of the dogs became anorectic and depressed. One of these dogs stayed overnight in the shelter and was found dead the next morning (Case 1). The second dog was taken into the owner’s family house for the night due to anorexia, intensive coughing and salivation (Case 2). According to the owner this dog had been vomiting blood during the night and demonstrated a rectal body temperature of 39.7°C. After a telephone consultation with the local veterinarian the owner administered 500 mg (19 mg/kg) of oxytetracycline (Oxytetral; Alpharma) orally and thereafter brought the dog to the small animal hospital at Norwegian School of Veterinary Science (NSVS) 140 km away, together with case 1 for post-mortem examination. The next day a third dog was found anorectic and depressed (Case 3) and was brought immediately to the NSVS. The rest of the kennelled dogs continued to cough, though no other animals developed further clinical signs. Cases 2 and 3 were more intensively trained than the other dogs on day 3 and 4 post-vaccination.

### Case 1

**Figure 1:**
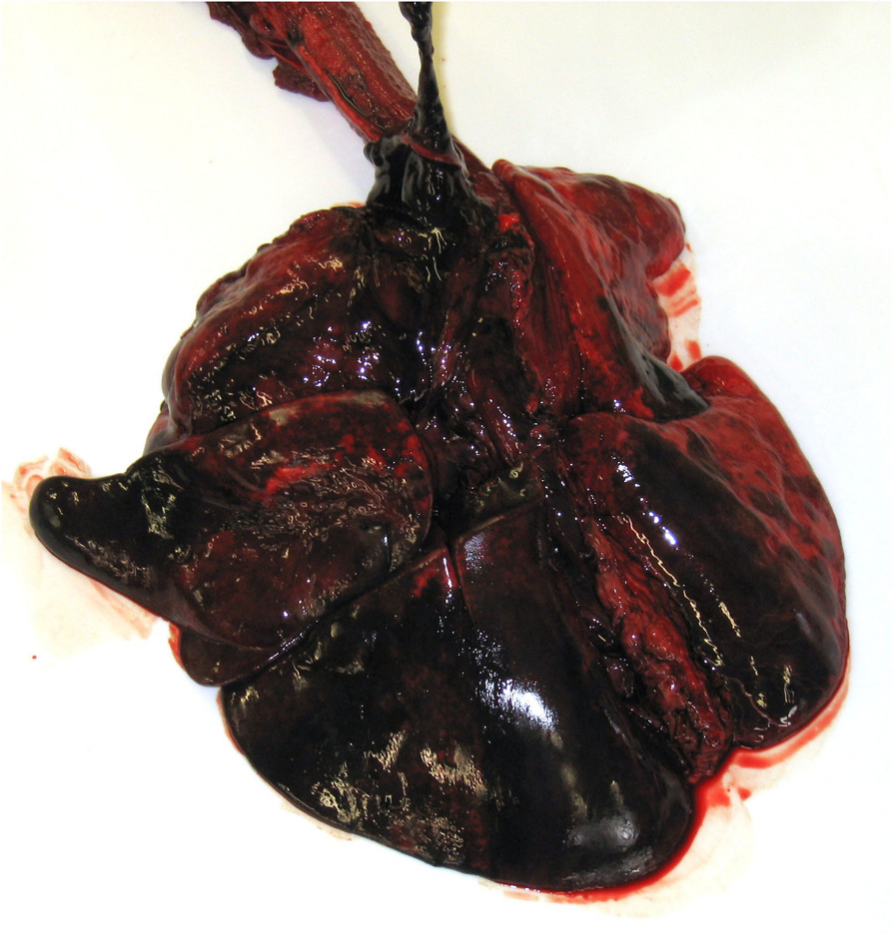
**Photographic overview of the lung, Case 1.** The lung failed to collapse and was haemorrhagic, most pronounced on the left side.

Post-mortem examination revealed epistaxis and haemorrhagic frothy fluids in the trachea and bronchial airways on cut sections. Haemorrhages were present in the thymus, epicardium, intercostally, and in the pleural space 200 mL of uncoagulated blood were present. The lungs were congested, wet, consolidated and diffusely to cavernous haemorrhagic, these changes being more severe in the left lung lobes (Figure 1).

**Figure 2:**
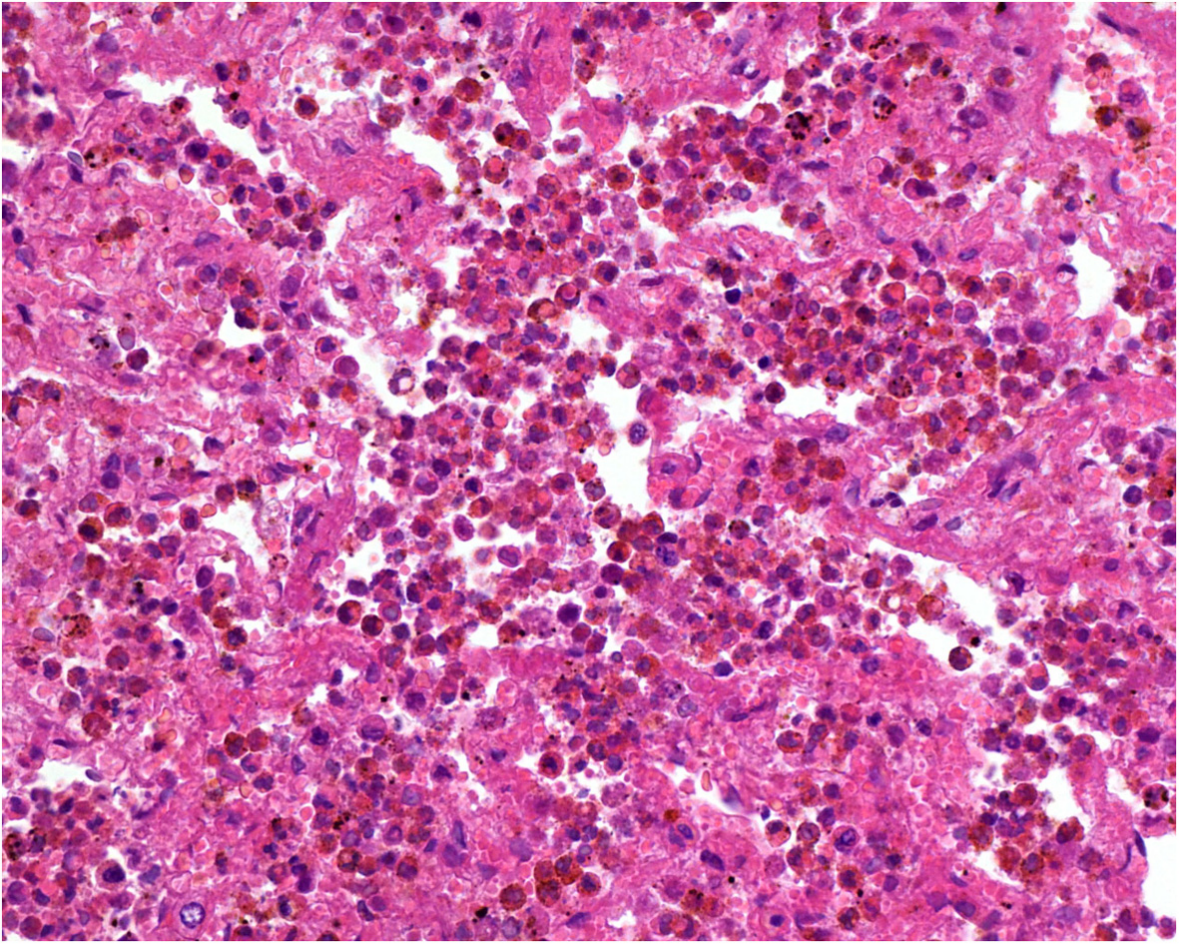
**Histological section of the lung, Case 1.** There is severe congestion and presence of inflammatory cells, erythrocytes and macrophages, which have engulfed red blood cells within the alveoli. HE stain, × 400.

**Figure 3:**
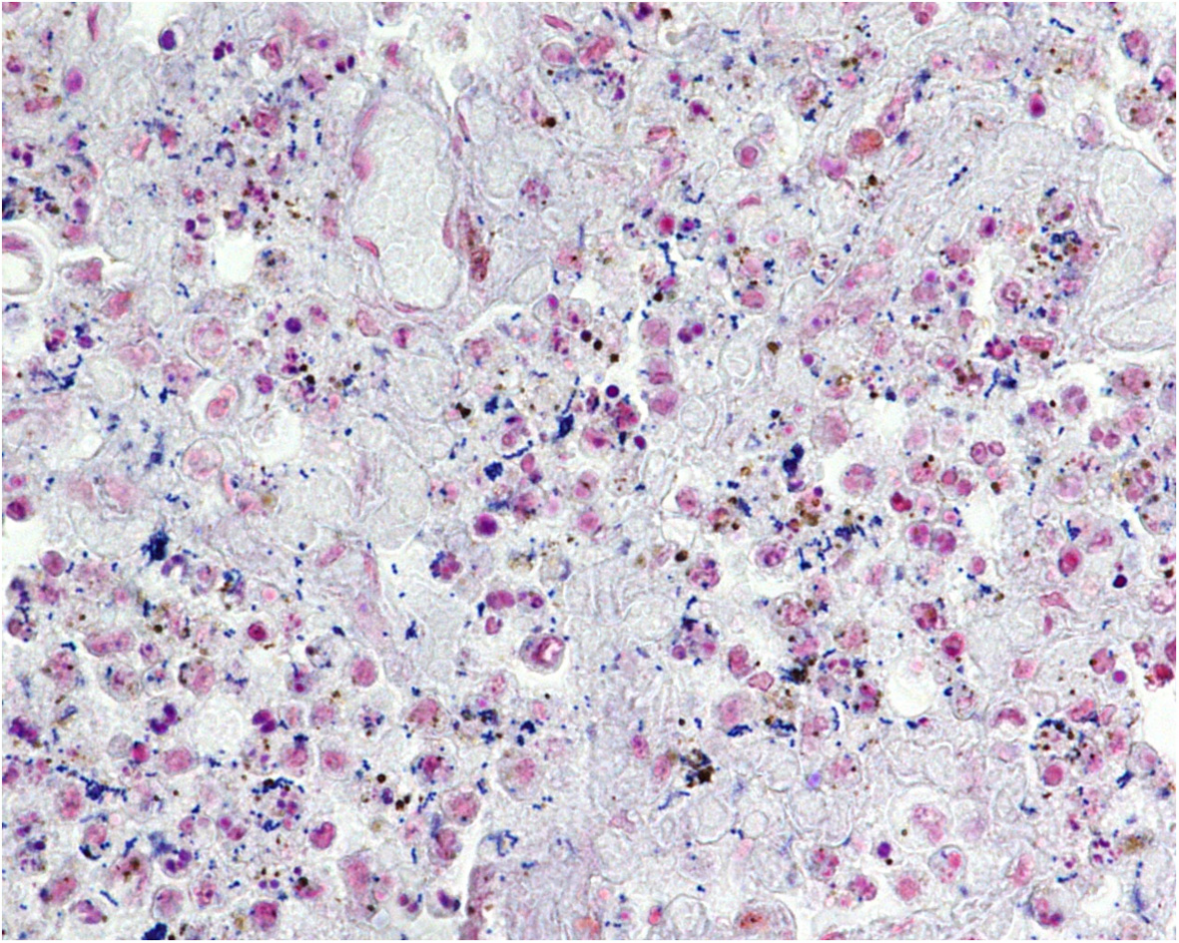
**Histological section of the lung, Case 1.** Gram-positive cocci are scattered within the alveolar lumina and also engulfed by alveolar macrophages. Gram stain, × 400.

Histopathology of the lungs revealed a subacute necrotising suppurative pneumonia, with haemorrhagic, often cavernous areas in the lungs and intra-lesional gram-positive cocci. A large number of macrophages with phagocytosed erythrocytes were present (Figures 2 and 3). A subacute pleuritis was also seen.

*Streptococcus equi* subsp. *zooepidemicus* was isolated in pure culture from the lung tissue with identification based on morphology, microscopy, Lancefield grouping (Streptococcal grouping kit, *Oxoid*, Basingstoke, Hampshire, England) and biochemical testing. Properties included β-haemolytic colonies on bovine blood agar, gram-positive, katalase negative cocci belonging to Lancefield group C. Glucose, lactose, and sorbitol were fermented, trehalose was not. The isolate was also tested by using API20STREP (bioMérieux^®^, Lyon, France) with the same conclusion.

Toxicological diagnostic screening of the liver tissue showed no evidence of anticoagulant poisons.

### Case 2

Clinical data for cases 2 and 3 are present in Table 1.

**Table 1:** Clinical data and symptoms for the three cases at admission to the hospital

**Case**	**Gender (intact)**	**Bred**	**Age (years)**	**Body weight (kg)**	**Body temp (°C)**	**Heart rate (beats/min)**	**Respiration rate/min**
1	Male	Mix-bred	3	23.5	n.a.	n.a.	n.a.
2	Female	Mix-bred	4	26.0	40.7	100	70
3	Female	Mix-bred	4	27.0	40.5	100	40

On presentation, the dog was depressed, dehydrated, shivering, hypersalivating with blood stained saliva, and coughed spontaneously with haemorrhagic expectorate. The neck was slightly stretched and auscultation of thorax revealed increased vesicular sounds.

**Figure 4:**
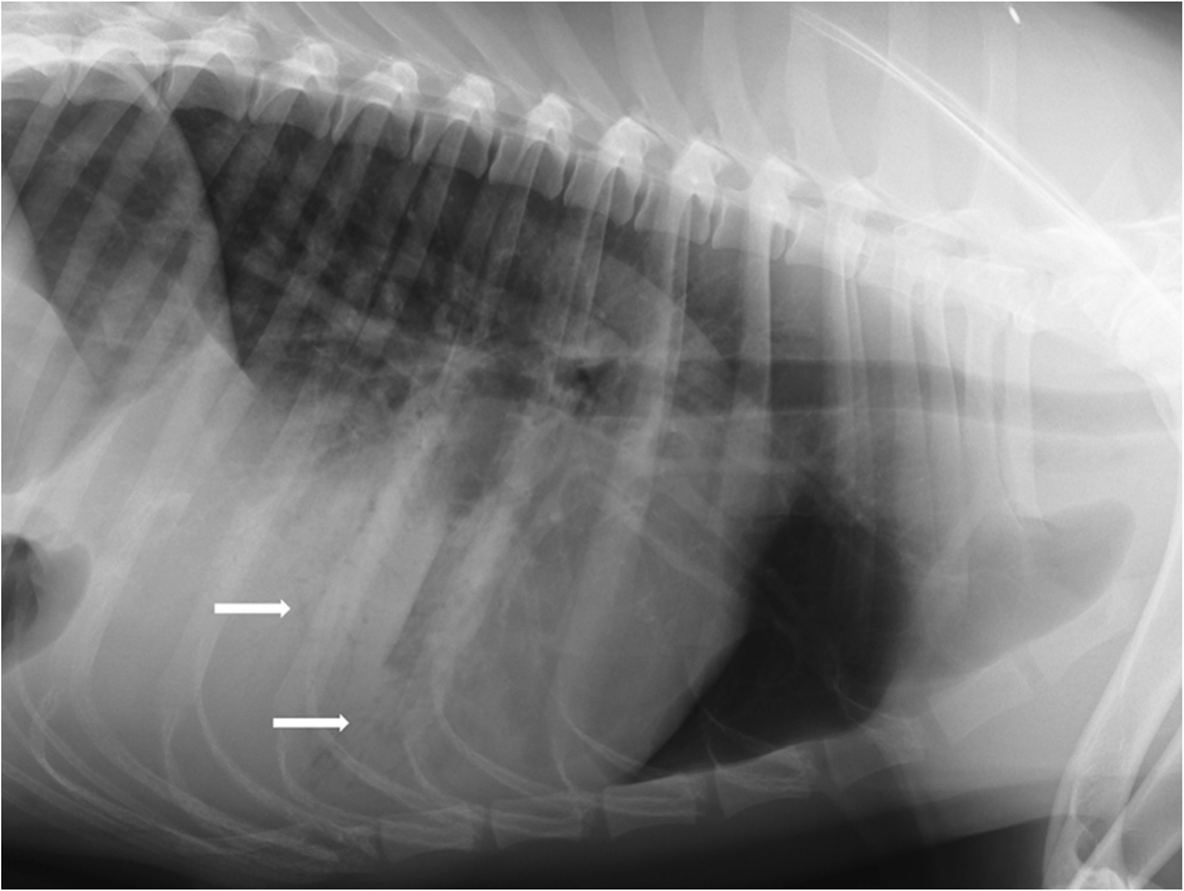
**Left lateral radiograph of the lung of case 2, first day of admission to hospital.** The radiograph reveals marked increased attenuation of the ventral part of the right caudal lung lobe and lobus accessorius, and moderate increased attenuation of the right middle lunglobe, with air bronchograms (white arrows).

**Figure 5:**
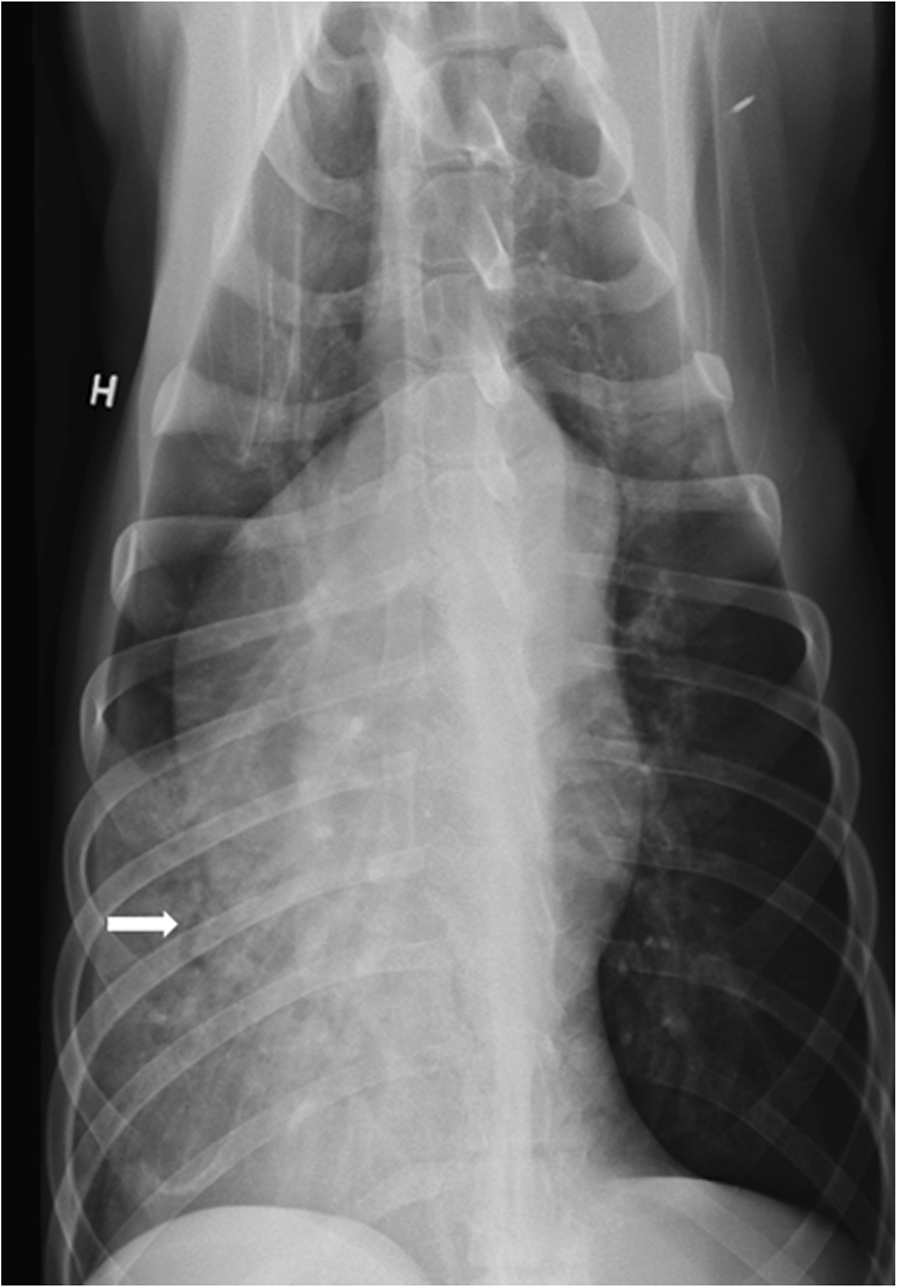
**Ventrodorsal radiograph of the lung of case 2, first day of admission to hospital.** This view also demonstrates the increased attenuation of the right middle, caudal and accessory lung lobes, with air bronchograms (white arrow).

Thoracic radiographs showed moderately increased attenuation of the ventral part of the right middle lung lobe, moderately to severely increased attenuation of the ventrocaudal part of the right caudal lung lobe as well as air bronchograms (Figures 4 and 5). These changes are consistent with acute pneumonia. A faint soft-tissue opacity was seen in the lung fissures, interpreted as a possible low amount of free pleural fluid.

The dog was treated with intravenous (IV) ringer acetate 100 ml/kg/hr for eight hours, thereafter 50 ml/kg/hr, enrofloxacin (Baytril, vet. Bayer; 5 mg/kg bodyweight (BW) IV once daily), ampicillin (Pentrexyl, Bristol-Myers Squibb; 35 mg/kg BW IV three times daily) and buprenorfin (Temgesic, Schering-Plough; 0.02 mg/kg BW IV three times daily). All medications were given for four days. The dog was hospitalised in an oxygen cage. Simultaneously as the treatment was initiated an expectorate sample was sent for routine bacteriological cultivation. *S. equi* subsp*. zooepidemicus* was isolated in pure culture and directly demonstrated to belong to the Lancefield Group C of streptococci. Complete blood count with serum biochemistry analysis was normal except for a mild leucopenia (Table 2). The coagulation profile was normal.

**Table 2:** Hematology parameters initially and in the rehabilitation period

**Parameter (*unit)***	**Case**	**Day 1**	**Day 4**	**Day 8**	**3 Weeks**	**5 Weeks**	**Reference **^**1**^
RBC *(× 10*^*12*^*/L)*	2	7.46	5.87	**4.94**	6.25	7.06	5.1 – 8.5
	3	5.71	n.a ^2^	5.78	6.67	6.45	
Hemoglobin *(g/l)*	2	**193**	149	125	157	178	120 – 180
	3	138	n.a	140	164	159	
HCT *(L/L)*	2	0.54	0.43	0.36	0.45	0.51	0.35 – 0.55
	3	0.40	n.a	0.40	0.47	0.46	
WBC *(× 10*^*9*^*/L)*	2	**3.3**	12.1	9.6	8.8	6.6	6.0 – 18.0
	3	**25.7**	n.a.	7.9	8.8	9.2	
Neutrophils *(× 10*^*9*^*/L)*	2	**3.0**	8.8	5.9	5.8	4.4	3.6 – 13.0
	3	**21.3**	n.a.	4.3	6.0	6.8	
Lymphocytes *(× 10*^*9*^*/L)*	2	**0.2**	1.1	2.0	2.1	1.6	0.8 – 5.8
	3	1.2	n.a.	2.0	2.0	1.6	
Monocytes *(× 10*^*9*^*/L)*	2	0.1	**2.8**	1.1	0.2	0.2	0 – 1.6
	3	**2.3**	n.a.	1.3	0.3	0.4	
Eosinophils *(× 10*^*9*^*/L)*	2	0.0	0.0	0.3	0.8	0.4	0 – 1.8
	3	0.0	n.a.	0.2	0.4	0.2	
Basophils (*× 10*^*9*^*/L)*	2	0.0	0.0	0.0	0.0	0.0	0 – 0.4
	3	0.0	n.a.	0.1	0.0	0.0	

^1^Reference ranges according to the Norwegian School of Veterinary Science’s Central Laboratory.

^2^*N.a.* not available.

Bold numbers are outside the reference frame.

On the second day of hospitalisation the dog showed substantial clinical improvement and was normothermic (38.1°C), less dyspneic, less tachypneic (respiration rate (RR) = 44/min), and had reduced salivation and coughing. There was no longer blood in the saliva nor epistaxis. The result from the bacteriological investigation of the expectorate demonstrated sensitivity against penicillin, tetracyclin, cefalexin, ampicillin, amoxicillin/clavulanate, enrofloxacin and linkomycin. The antibiotic regimen was switched to phenoxymethylpenicillin (Apocillin; Actavis) 660 mg per os (PO) three times daily for 14 days. No diagnostic tests for respiratory viruses were performed. Clinical signs gradually resolved over the next few days and the dog was sent home seven days after hospitalisation. Control radiographs before departure from the clinic revealed absence of air bronchograms, though a mild to moderate increased attenuation with an interstitial pattern was still present.

### Case 3

This animal was presented to NSVS one day after Case 1. The dog had been coughing for several days and gradually worsened with reduced appetite and depression developing on the day of presentation. On physical exam there was moderate dyspnoea with abdominal respiration and increased vesicular sounds, slight neck extension, blood stained saliva as well as fever (Table 1). Haematology showed moderate leucocytosis due to neutrophilia and monocytosis (Table 2). Radiography of the thorax showed the same changes as described for Case 2.

The dog was hospitalised and medically treated in the same way as for Case 2. Clinical progression was similar to Case 2, with normalisation of temperature (38.8°C), respiration rate (28 breaths/min), heart rate (100 beats/minute) and appetite on the second day of hospitalisation. No salivation or spontaneous coughing was observed unless whilst excited after visiting the exercise pen. Repeat thoracic radiographs on the seventh day of hospitalisation revealed air bronchograms in the right middle lung lobe, but reduced consolidations. The dog was sent home on phenoxymethylpenicillin (Apocillin; Actavis) 660 mg PO three times daily for another 14 days.

**Figure 6:**
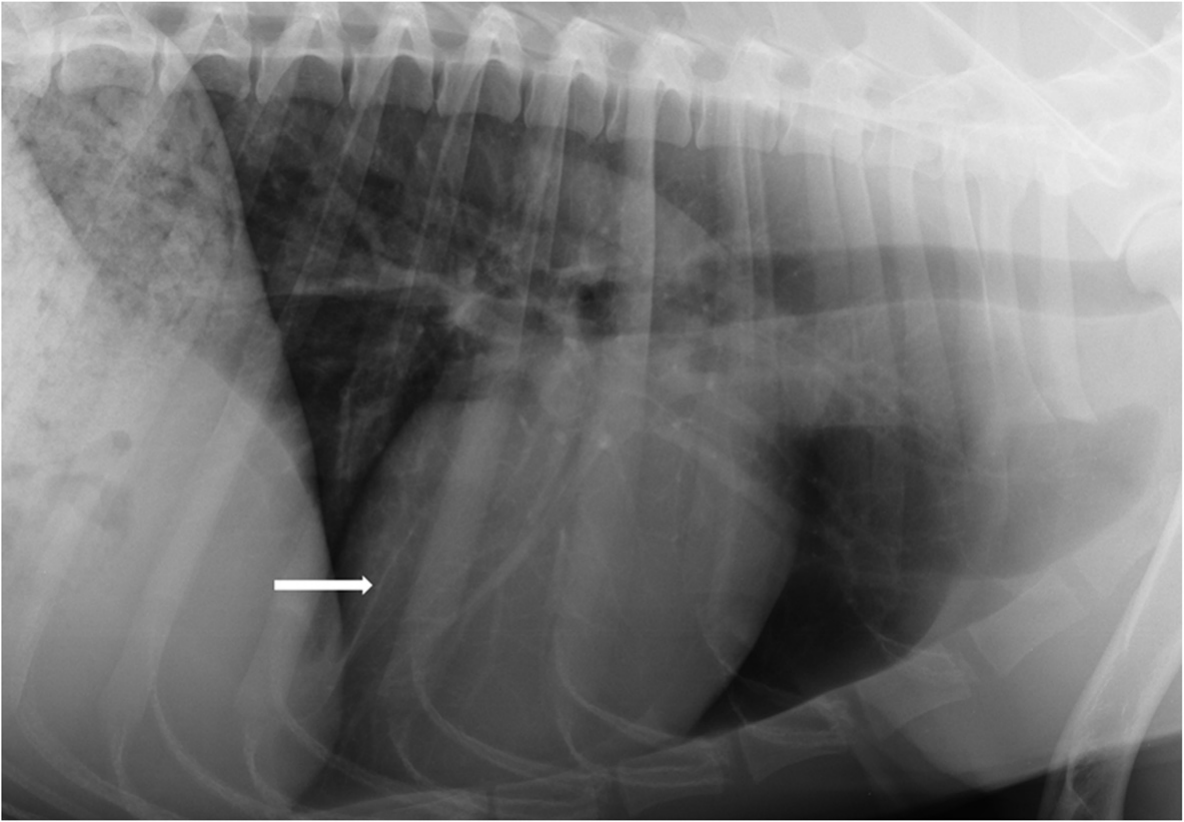
**Control radiograph of the lung of case 2 eight weeks after admission to hospital.** Left lateral radiograph reveals a faint line (white arrow) which is interpreted to represent either mild amount of pleural fluid or mild thickening pleura.

**Figure 7:**
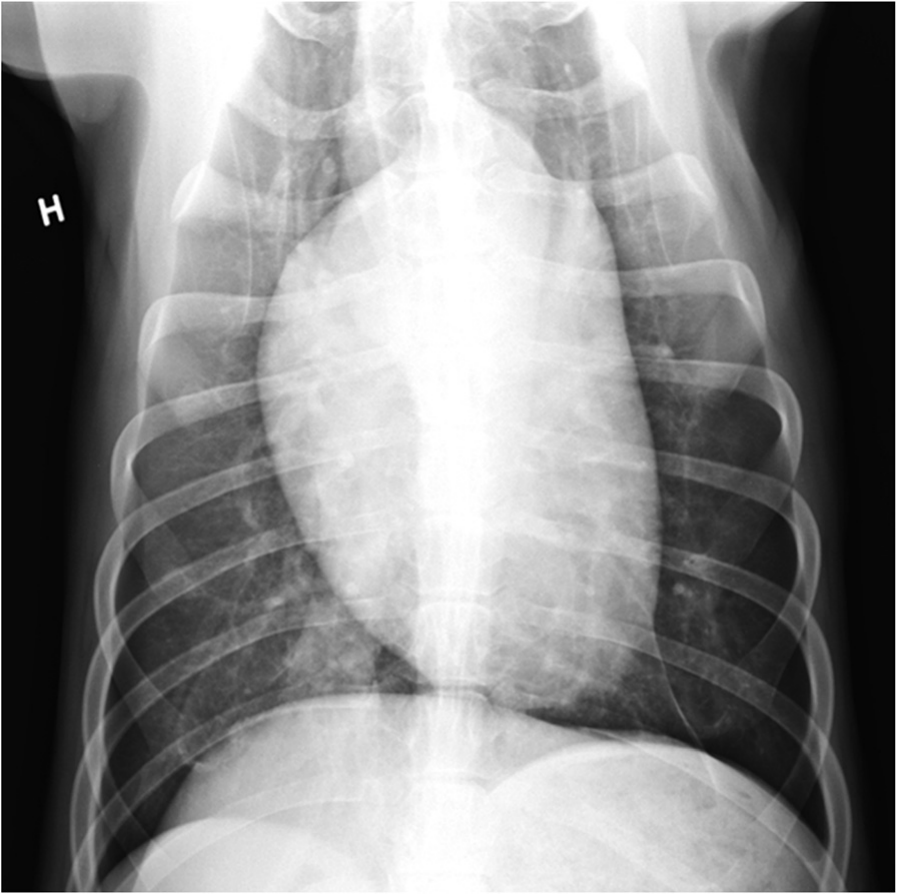
**Control radiograph of the lung of case 2 eight weeks after admission to hospital.** Ventrodorsal view reveals a mild, mild increased attenuation in the right caudal lung lobe, which was interpreted as likely mild “scar tissue” from the previous severe pneumonia.

Follow-up hospital care of both dogs included radiographs of the thorax after one, three, five and eight weeks (Figures 6 and 7) together with complete blood counts (Table 2). After thoroughly scrutinising the last taken radiographs together with assessing their clinical condition they started a step-wise training program.

The radiographs taken during recovery revealed a very mild interstitial attenuation of the lung lobes that had been most severely affected and mild to faint visualisation of fissure lines which was interpreted as either mild amount of free fluid or mild fibrosis. These minor findings were gradually reduced, but faint fissure lines could still be seen after five weeks for Case 3 and after eight weeks for Case 2 (Figures 6 and 7).

The dogs were kept confined for one week after they were released from the hospital, and did run free on a large (2–3 acres) fenced yard for two weeks. Case 2 was in full training eight weeks post infection and Case 3 was slightly behind.

Early in January the following year both dogs participated in a sled race resulting in a time track record, and medal placements in various championships were achieved the following season.

## Discussion

The dogs in this highly trained sled-dog pack competed in a sledge dog race after prolonged transportation and were additionally moderately exercised before the race. Three days after the sled race the dogs were given a commercial polyvalent canine vaccine containing CDV, CAV-2, CPV, CPIV, CCoV and leptospira. Clinical signs of respiratory disease were observed four days after vaccination. Immunosuppression in dogs immunised with a similar polyvalent vaccine was reported by Phillips and others [9]. They demonstrated that the absolute lymphocyte count in blood was suppressed on day five and seven following immunisation and that one group of dogs immunised with CDV and CAV-2 also had decreased lymphocyte count on day three post vaccination. All the dogs produced neutralising antibodies to canine distemper virus and some parts of the natural immunity were not influenced by the vaccination. However, the immunisation with polyvalent vaccines significantly suppressed the lymphocyte response to phytohaemagglutinin, a mitogen that induce proliferation in T-lymphocytes in vitro on day 5, 7 and 11 post inoculation, but returned to normal response level by day 14 following immunisation. The combination of CDV/canine adenovirus type 1 (CAV-1) or CDV/CAV-2 was believed to be responsible for the suppression in lymphocyte responsiveness. The duration of the suppressed lymphocyte response was at least seven days and was most pronounced on day 7 post inoculation with up to 90% suppression of mitogen-induced lymphocyte response compared to control dogs. In the present study, the use of modified live polyvalent canine vaccine combined with stress associated with training, competition and prolonged transportation may have suppressed the innate immune response to *S. equi* subsp. *zooepidemicus,* which seldom causes pathology in dogs. The possibility of a co-infection with viral pathogens cannot be excluded and may have contributed to this outbreak.

Outbreaks of haemorrhagic pneumonia in dogs due to *S. equi* subsp. *zooepidemicus* have been described following transportation to a race track associated with sudden change in the weather [8], in newly arrived densely housed research dogs [4,6,7] and in kennelled dogs [3,10-12]. In most of these reports the cause of the outbreak of CIRD is believed to be primary viral agents alone or in combination with environmental factors that induce secondary peracute pneumonia. Some of these papers report the use of CDV/CAV-2 vaccine prior to infection with *S. equi* subsp. *zooepidemicus*[3] or a possible dual infection with CDV [4], though do not postulate that immunosuppression due to vaccination with modified live polyvalent canine vaccine is significant in this pathogenesis.

In a study of experimental parvovirus infection in dogs, Potgieter and others [13] observed that dogs vaccinated with modified live CDV and CAV-1 five days before challenge with virulent canine parvovirus resulted in disease caused by canine parvovirus whereas unvaccinated dogs remained healthy. This may indicate that these animals had reduced immunity associated with recent vaccination.

Histopathologically, the pneumonia in Case 1 was severe with the presence of inflammatory cells, leakage of blood into the lung tissue, and intralesional gram positive cocci, which were likely to be *S. equi* subsp. *zooepidemicus (*Figures 2 and 3)*.* Similar histopathological findings were also described in the paper of Priestnall and others [14], who specified that *S. equi* subsp. *zooepidemicus* is associated with acute and often fatal clinical disease in dogs.

A possible source of *S. equi* subsp. *zooepidemicus* in the outbreak described in this paper was neighboring horses. However, there may have been sub-clinical carrier animals within the pack as Chalker and others [3] isolated this pathogen from 9.7% of clinically healthy kennel dogs, but where the kennel had a history of endemic CIRD.

*S. equi* subsp. *zooepidemicus* is not normally carried by dogs, but it can probably cause disease in a situation where there is high level exposure from diseased dogs or other sources in the environment, an on-going viral infection, temperature stress, transport stress, intense exercise associated with training and competition and vaccine induced immunosuppression. In a retrospective study of 393 cases of streptococcal infections in dogs, four cases (1%) were found to be caused by *S. equi* subsp. *zooepidemicus*[15]. In humans, *S. equi* subsp. *zooepidemicus* represents a potential virulent zoonotic pathogen, with infection inducing a toxic shock syndrome following contact with horses [16-18].

One report used a study population from a re-homing shelter with an endemic respiratory disease, including 39 dogs positive for *S. equi* subsp. *zooepidemicus* culture on post-mortem lung wash and 16 negative control dogs. An objective scoring system based on histopathological lung examination demonstrated that 26 (67%) of dogs with a positive culture had evidence of pneumonia caused by *S. equi* subsp. *zooepidemicus*[14]. The lungs of the dogs affected with severe pneumonia caused by *S. equi* subsp. *zooepidemicus* had significantly elevated levels of expression of the genes of pro-inflammatory cytokines indicating a strong innate immune response caused by the invading pathogen.

Paillot and others [19] demonstrated that three novel genes (*szeF*, *N* and *P*) for production of superantigens in *S. equi* subsp. *zooepidemicus* are occurring in half of the isolates from cases of disease. The authors indicate that the introduction of these genes to the population of *S. equi* subsp. *zooepidemicus* may be the cause of an emerging trend of severe pneumonia caused by this pathogen. However, in the study of Priestnall and others [14] there was no indication that the carriage of these superantigen encoding genes is related to the severity of the histopathological changes. The authors emphasise that further knowledge and alertness of early clinical signs will contribute to enhance the treatment success and avoid transmission of the causative strain of *S. equi* subsp. *zooepidemicus* to other animals and humans.

The rate of resolution of pneumonia in humans has been defined as the resolution of radiographic abnormalities associated with the disease [20]. Patients with moderately severe pneumonia have a radiological clearance rate of 70% after 1 month, and more delayed if the pneumonia is severe [21]. A multicenter cohort study that included adult patients classified as at low risk of short-term mortality concluded that full resolution of symptoms from community-acquired pneumonia (CAP) may take more than 28 days [22]. This study was not specific to athletes, as they are thought to have a better physical health status compared with the general population. When to recommence training for athletes following pulmonary infection depends upon the underlying microbial aetiology and should be individualised [23]. There is no reason to believe that dog athletes should be advised differently. The long-term rehabilitation of high performance sled dogs may be complicated because of the dual challenge of strenuous exercise and low temperature.

As a general rule, caution should be taken to prevent athletes from returning to competition too fast. In human athletes, potential complications such as pneumothorax, bronchiectasis and hemoptysis has been recorded, as well as acute respiratory failure. Furthermore, intense exercise too early in the rehabilitation process can increase susceptibility to viral illness and weaken the muscle performance [24].

We may speculate that the environmental stress related to the transportation, high-end competition, time of vaccination and too early exercise all contributed to the immunosuppression and consequently the susceptibility to virus- and/or bacterial infection, and where potentially more aggressive and opportunistic bacteria like *S. equi* subsp. *zooepidemicus* can progress quickly. It may also be advisable to choose a later moment for vaccination than close up to a challenging physical competition, and to avoid any strenuous exercise for at least ten to 14 days after immunisation.

Return to play is a hot issue within the top-level sports, but without any specific guidelines post pneumonia. These dogs were in a very good physical shape at the start of the outbreak of haemorrhagic pneumonia, and the two surviving individuals recovered relatively fast and returned to their previous performance level by setting time track records in their first race after recovery.

## Conclusions

Environmental stress associated with intense exercise, competition and prolonged transportation combined with canine vaccination may suppress the innate immune response to viral infections and subsequent *S. equi* subsp. *zooepidemicus* infection that only rarely cause pathology in dogs. Knowledge and alertness of early clinical signs of acute haemorrhagic pneumonia will enhance treatment success and shorten the rehabilitation period. However, caution should be taken to prevent athletes from return to the competition arena too fast. These high performance sled dogs recovered relatively fast and could prove their fitness by competing and winning international sled race championships three months later.

## Abbreviations

CPIV: Canine parainfluenza virus; ITB: Infectious tracheobronchitis; CIRD: Canine infectious respiratory disease; CAV-1: Canine adenovirus type 1; CAV-2: Canine adenovirus type 2; CDV: Canine distemper virus; CPV: Canine parvo virus; CCoV: Canine enteric coronavirus; CAP: Community-acquired pneumonia; NSVS: Norwegian School of Veterinary Science.

## Competing interests

The authors declare that they have no competing interests.

## Authors’ contributions

GJ did the clinical examinations, drafting and revising the manuscript. . HKS evaluated the radiographs initially and the controls. ØK did the postmortem examination. BB and HS did the microbiology testing. HJSL and HS made substantial contribution to the discussion in the manuscript. All authors read, revised and approved the final version of the manuscript.
